# A Qualitative Investigation of Staff Feedback on an Online Learning Module on Smoking Cessation in a German Healthcare Company

**DOI:** 10.3390/healthcare11121774

**Published:** 2023-06-16

**Authors:** Karin Vitzthum, Deniz Cerci

**Affiliations:** 1 Institut für Tabakentwöhnung und Raucherprävention, Vivantes Netzwerk für Gesundheit GmbH, 13407 Berlin, Germany; 2 Klinik für Forensische Psychiatrie, Universitätsmedizin Rostock, 18147 Rostock, Germany

**Keywords:** smoking cessation, nicotine dependence, online learning, staff feedback, healthcare services

## Abstract

Quitting smoking is a powerful way for patients to improve their own wellbeing and to significantly reduce the risk of health problems. Evidence shows that health professionals can effectively intervene in order to prevent and stop tobacco smoking in their patients. Online learning modules have proved to be effective in terms of transferring knowledge and skills. In an urban community hospital setting in Germany, a novel e-learning course for staff on the treatment of tobacco dependence was implemented in 2021. In this study, we analyzed free-text feedback of participants completing this online module in order to examine the feasibility and acceptance of this new format. We were able to reach a reasonable proportion of staff. Our qualitative analysis showed that most feedback was positive and described the module as well-designed and helpful. Some staff, however, expressed extremely negative views and did not see smoking cessation support as essential to their role in healthcare. We argue that in order to achieve a shift in attitude in healthcare staff, a change in German policy is required which includes the creation of smoke-free environments and the adherence to smoke-free policies on hospital sites. Furthermore, the provision of smoking cessation support in line with the WHO Framework Convention on Tobacco Control and a true understanding of the role of all healthcare professionals in promoting health of patients and staff will be essential.

## 1. Introduction

Tobacco smoking is still the most dangerous, widespread but also avoidable cause of various diseases and premature death [1]. Quitting smoking is a powerful way for patients to improve their own wellbeing, to reduce the risk of health problems, and to minimize complications of surgical and medical procedures, e.g., oncologic treatments [2,3,4]. While being admitted to hospital offers a teachable moment to approach this issue, many healthcare workers ignore or avoid this topic when talking to patients, possibly due to the fact that they smoke as well or because they have tried to help patients quit smoking in the past but were unsuccessful [5,6,7]. Additionally, smoking cessation counselling is not compensated financially by health insurance companies in Germany [8]. Under current circumstances in the healthcare system, time pressure, high workloads, and administrative burdens thwart prevention and health promotion in favor of high-end medical procedures [9].

In Germany, approximately 16 million people are current smokers, and every year 127,000 people die from the health effects of smoking [10]. When comparing tobacco control measures in Europe, Germany comes close to last place [11]. This indicates that there has not been enough societal norm change that supports policy approaches to reduce tobacco use. This also applies to German healthcare settings, in which the implementation of smoke-free environments and the provision of smoking cessation services falls behind its neighbors in Europe, as staff show reluctance to implement changes [12]. In the recent past, however, quality management indices in Germany have focused more on tobacco cessation than previously. One example is the introduction of a German certificate as a cancer center which also requires hospitals to offer smoking cessation services [13].

Evidence shows that health professionals can effectively intervene in order to prevent and stop tobacco smoking in their patients [14,15,16]. Individuals who smoke are more likely to quit when advised to do so by a healthcare professional [17]. The lack of knowledge and skill has been identified by health professionals as a barrier which can only be overcome by adequate training [18,19,20]. Digital training for healthcare staff in the field of smoking cessation has been shown to be as effective as common offline learning methods [21,22,23]. German medical staff, however, are generally trained poorly on how to help patients to stop smoking [24,25].

The update to the relevant German guideline on smoking cessation, the S3 guideline [8], provided an opportunity to design and implement a novel e-learning course on treating tobacco dependence for staff in a state-owned healthcare company in an urban German environment. We used this occasion to carry out a qualitative analysis of the optional feedback section at the end of the training module in order to examine the feasibility and acceptance of this new format. The aim was to explore views on staff training on smoking cessation support and to explore opportunities and barriers in the process of its implementation.

## 2. Methods

In this study, we carried out a qualitative analysis of free-text responses to an open question asking for optional feedback on an online learning module on smoking cessation.

### 2.1. Setting

The investigation was carried out at a German state-owned healthcare company which runs eight hospitals with a variety of inpatient and outpatient facilities. Smoking is officially prohibited but designated smoking areas exist on hospital sites. The company employs more than 15,000 staff members. All staff are required to attend an induction program in the first weeks after taking up employment and to complete a number of online learning modules on a regular basis during the course of their employment.

### 2.2. Learning Module

A new online learning module on smoking cessation was introduced in order to increase staff competence in smoking cessation treatment and adherence to the relevant German S3 guideline. The module was designed with the help of the IT department by using the software “articulate 360”. Its content was created by the first author (KV) based on a presentation on the same topic, which had been part of the induction program until then. The module consists of about 30 slides and can be completed within 15–20 min. It includes interactive and multimedia elements and covers the diagnosis and treatment of tobacco dependence according to the German S3 guideline. The users are presented with a case vignette of a lorry driver being admitted to hospital with sleep disturbance, dyspnoea, and cough. His physician explains diagnostic and treatment options regarding smoking cessation over the course of the patient’s stay in hospital and thereby introduces the user to the following topics: the pathophysiology of smoking; the diagnostic criteria of tobacco dependence in ICD-10; the Fagerström-Test; carbon monoxide monitoring; options for prevention; intervention and treatment (self-help resources, motivational interviewing, brief advice interventions, nicotine replacement therapy and medication); and specific requirements for documentation.

### 2.3. Participants

All participants were adults aged 18 and above. They were employees completing a series of mandatory online modules for staff training, the smoking cessation module being amongst them. The module provided an opportunity to give feedback using a free-text box at the end of the training. All participants were provided with information to enable them to make an informed decision on whether to provide feedback or not; they did not have to provide feedback if they did not wish to. Due to the anonymous completion of the survey, once the responses were submitted it was not possible for the data to be withdrawn. All responses were included in the qualitative analysis.

### 2.4. Data Collection

Convenience sampling was undertaken. Data were gathered between June 2021 and May 2022. Anonymized responses were transferred from the secure database via Microsoft Excel.

### 2.5. Analysis

We applied inductive thematic analysis, an approach which derives coding and themes from the raw data [26,27,28]. This approach is well-established in the field of qualitative free-text survey analysis [29,30,31].

An analysis of the data was carried out using a structured approach. In the first stage of analysis, two independent researchers (KV and DC) familiarized themselves with the data. They read the free-text comments several times, in order to gain a good understanding of the content, quality, and depth of the comments. They then coded the data accordingly to describe the nature of the comments. The responses were first sorted into first-order codes, based on whether the comment was (1) positive, (2) negative, (3) neutral, (4) both positive and negative, or (5) neither positive nor negative nor neutral.

In the second stage, the researchers applied second-order codes which had been derived inductively from the data. In total, eight second-order codes were assigned. Comments were given as many codes as appropriate to the content. The researchers discussed discrepancies and disagreements; these were resolved through a process of consensus. Similar codes were amalgamated. The comments were grouped by second-order code and compared. The researchers then identified the themes derived from the data. Codes and themes were measured in order to give an indication of their prominence.

To improve the credibility of the qualitative analysis, the researchers engaged in reflective discussions and reviewed the data and the identification of codes and themes multiple times to ensure that they reflected the raw data accurately and honored the perspectives of the participants.

All answers were provided in German. For the purpose of this article, the responses were translated literally/verbatim to guarantee an authentic impression as much as possible.

Due to the feedback being anonymous, we are not able to allocate the comments to specific professional groups or hospital sites.

## 3. Results

Within the first 12 months of launching the learning module we reached an average of 82% of all employees (N = 6935), out of which almost 2% (N = 119) used the option to comment on the unit.

The professional background of all participants is shown in Table 1.

**Figure 1:**
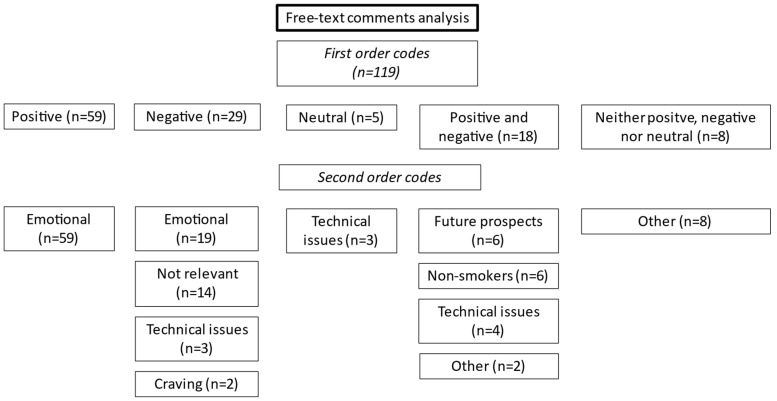
Free-text comments analysis of staff feedback on an online learning module on smoking cessation.

First-order codes show more positive (*n* = 59) than negative (*n* = 29) comments, with a ratio of almost 2:1 (Figure 1). A group of participants provided positive and negative feedback in their response (*n* = 18), whereas only a few gave feedback which was neutral (*n* = 5) or neither positive, negative, nor neutral (*n* = 8).

### 3.1. Positive

- “Extremely informative (even though I (as an ex-smoker) thought of myself as very knowledgeable); I was surprised to learn that quit attempts are not considered as symptoms and that hypnosis is not an effective therapeutic intervention.”
- “I think this unit is very well-designed and diversified for its varied media content.”
- “A very entertaining and helpful unit—many thanks.”
- “The unit is designed beautifully—the best on this platform—thanks.”
- “Great input! … The structure the unit follows is very motivating!”

### 3.2. Negative

- “Making this online module compulsory is not justified by legal requirements and, in my view, is an arbitrary measure with which the employer only intimidates staff and misuses their working hours.”
- “Absolutely pointless training. As the next topic, I suggest further compulsory training, in which I ask all employees about the focal points of my work, including ICD coding of posterior shoulder instability. Complete waste of time!”
- “This is going much too far; the content should not be part of this online programme, this belongs to the postgraduate training of physicians and should not be compulsory; just keep it to the legal commitments—what will be next—a swimming course?!”

We then assigned all answers to eight categories (Table 2): emotional, not relevant, non-smokers, technical issues, craving, future prospects, and others. Please note that some comments were assigned to multiple second-order codes. The largest group, about half of all participants (*n* = 59), provided brief positive, often encouraging and what we would describe as mostly emotional feedback on the unit. Negative comments with an emotional connotation were often strongly worded, and sometimes condescending or cynical (*n* = 19). A group of participants (*n* = 14) felt that the module was not relevant to their work or expressed anger about having to complete it. Similarly, some non-smokers felt that they should not be asked to complete the module (*n* = 6). Comments raising technical issues criticized or raised technical problems with completing the module (*n* = 10). Some participants pointed out that the module triggered nicotine craving (*n* = 2), whereas others talked of future prospects suggesting new ideas to improve smoke-free policies (*n* = 6). Non-sensical or irrelevant feedback was assigned to the category “other” (*n* = 10). Corresponding themes to second-order codes are listed in Table 2. The corresponding themes are not verbatim responses taken from the comments but recurring themes which could be elicited from the answers.

### 3.3. Not Relevant to Job Role

- “Are you serious? What else are we supposed to do? Have you ever heard of the situation with staff shortages? It is enough! This should be done by doctors when patients are admitted.”
- “I am not planning to persuade elderly people of stopping smoking; I also doubt that this unit should be mandatory, I do not accept this as part of my job role—unless a patient asks me of their own volition.”
- “Since when have nursing staff been allowed to and obligated to carry out therapeutic interventions? There is no training for this! Nothing can happen to us as long as “highly qualified” doctors are available!”

### 3.4. Non-Smokers

- “As a non-smoker and a non-educator, this online module was simply impossible for me.”
- “It is completely unacceptable to bother me—a non-smoker—with something like this and take up valuable time that I could spend with patients.”
- “Not required for non-smokers.”

### 3.5. Technical Issues

- “It would be nice to be able to print out interesting passages.”
- “I needed more than 10 min to complete this module.”

### 3.6. Craving

- “Hi, I have been smoke-free for 3 years. And it was a tough fight. I have to admit that this module was difficult for me, not because of the questions, but the term “smoking” was used so often. Now I have to try to refocus and distract myself. It would not be a bad idea to warn ex-smokers to be cautious because of involved triggers. Thank you.”
- “I am right in the middle of quitting and feel indeed through this mandatory unit, which talks permanently about smoking (including a picture of a lit cigarette), quite triggered. Very well done! Thank you very much for this.”

### 3.7. Future Prospects

- “A suggestion for promoting the health of staff which is common practice in the Helios Group: one additional day of annual leave for all non-smokers”
- “I support a complete smoking ban which is strictly implemented!”

## 4. Discussion

We qualitatively assessed free-text feedback on an online learning module on smoking cessation which is, to our knowledge, the first of its kind to teach smoking cessation interventions to healthcare staff in Germany. Due to its mandatory characteristic, we were able to reach a reasonable proportion of our staff. The free-text nature of the feedback provided participants with the opportunity to expand on aspects which they deemed relevant.

The analysis of the comments revealed that the ratio of positive to negative feedback was 2:1, which shows that the majority of participants supported the format and found it helpful to learn how to treat smokers. However, the analysis shows a high proportion of emotional responses and negative feedback, with many members of staff feeling that the content was not relevant to them or that smoking cessation is not part of their job description. We were at times struck by the extremely negative and cynical connotation of some comments, especially when healthcare staff showed no appreciation of the importance of smoking cessation whatsoever or could not see any role for themselves in encouraging patients to quit.

A reason may be staff feeling stressed and overburdened by taking on a responsibility which they feel is not essential to fulfilling their core duties in clinical care. In particular, nursing staff have reportedly quit their jobs or reduced working hours, leading to many open positions that cannot be filled [32].

Online learning modules are helpful in delivering specific knowledge, but emotional roadblocks to transfer theory into practice need to be considered. Staff may not understand the role that they can play beyond providing cessation support to patients. Other factors beyond knowledge and skill have to be considered, which include being a role model by not smoking, and promoting compliance with smoke-free hospital policy [33].

The German healthcare system is known for maintaining a strict hierarchy. This may be a reason why many comments are concerned with “whose job it is” to help patients quit smoking. This is unfortunate, as an important learning opportunity irrespective of professional background may be missed [34,35].

Healthcare staff often show contradictory views with regard to the smoking status of their patients and their own smoking habit. Staff who smoke often harbor misconceptions about smoking [36], which is likely to make them less effective in providing smoking cessation support. Accepting a role model position for patients might cause them stress, as they may experience ambivalence about the need to stop smoking themselves but at the same time not feel affected by health-related concerns [37,38]. We believe that this may also partly explain the strongly worded comments of those participants who had an extremely negative view of the online module.

Interestingly, a small group of participants commented on pictures used for illustration causing them to experience cravings for nicotine. These images included smoke and burning cigarettes. The importance of cues in films and advertisements has been highlighted in the literature and is also relevant in this context [39,40,41,42,43]. We will pay attention to this very helpful feedback when updating the learning module. Furthermore, we will try to make the content more relevant by considering different professional groups and their roles in providing smoking cessation support.

We believe that a shift in attitude in staff can only be achieved by political measures. There is evidence that a change in attitude with regard to smoking can often be observed after appropriate measures have been introduced [44,45]. This is why we support the systematic implementation of the WHO Framework Convention on Tobacco Control, as we believe that appropriate policies and the creation of smoke-free environments will increase the acceptance of the task of treating nicotine dependence, particularly in healthcare settings. “A strategy for tobacco-free Germany 2040” [10] outlines ten measures in order to achieve a smoking prevalence of less than 5% in adults, e.g., by increasing tobacco taxes and by banning tobacco advertising. The provision of free nicotine replacement therapy and psychological interventions to support smoking cessation will also play a central role in this process. This shows that the education of healthcare professionals on this topic will continue to remain relevant and we view the mostly positive and constructive feedback as an encouragement to continue staff training in this field.

A strength of this study is the structured approach that was used to analyze the data set. We have been able to reach a significant proportion of staff, and the results show that a wide range of views and perspectives have been expressed and analyzed. The anonymous nature of the feedback may have enabled participants to provide honest responses without fear of reprisal.

However, qualitative research always implies subjective decisions about the identification of codes and themes. We attempted to minimize this effect by following a rigorous and transparent process. In order to preserve anonymity of the data and to increase the response rate, we only collected limited data on demographics. This meant that we were unable to determine if particular groups made negative comments more frequently, e.g., staff who smoke or work in roles that do not have direct patient contact. The format of a free-text box for feedback, and the high proportion of non-responders to the question of interest, limit the generalizability of our findings. Responders and non-responders may have views and attitudes, which, for different reasons, they did not express.

## 5. Conclusions

The qualitative feedback on an online learning module for smoking cessation in a German hospital setting shows that the majority of staff support this approach as a learning opportunity. However, some voice very negative views and do not feel that this topic is relevant or should be part of their role as health professionals. This highlights that the content of a learning module needs to be relevant and meaningful for different professional groups depending on the type of patient contact they have. Some participants have pointed out that images of smoke and cigarettes can trigger craving for nicotine, which should be taken into account when designing educational content. Furthermore, we believe that political measures to create smoke-free environments and to provide smoking cessation support will be essential to achieving a shift in attitudes in staff in the long term.

## Figures and Tables

**Table 1:** Proportion of professional groups having completed the online learning module on smoking cessation.

Profession	Completed	Not Started Yet	Started
Doctors	76.8%	22.1%	1.1%
Physiotherapists	81.4%	18.1%	0.5%
Laboratory assistants	80.2%	18.6%	1.2%
Nursing staff	83.7%	15.2%	1.2%
Specialized services	100.0%	0.0%	0.0%
Administration staff	45.4%	45.5%	9.1%
Total	81.5%	17.4%	1.1%

**Table 2:** First- and second-order codes in qualitative analysis of staff feedback.

Second-Order Code	Number of Responses (*n*)	Proportion of Responses (%)	Corresponding Theme
Emotional (positive)	59	49.6	“Thank you for the information.” “I learnt something new.” “This is more helpful than other mandatory modules.” “I like the design of the module.”
Emotional (negative)	19	16.0	“I should not have to do this.” “Patients should not be forced to give up smoking.” “Hospital management wants me to stop smoking.”
Not relevant to job role	14	11.8	“Helping patients to stop smoking is not part of my role.”
Technical issues	10	8.4	“I had technical problems completing the module.” “ I would like to have a printed copy of the module.” “I need more time to complete this module.”
Craving	2	1.7	“The module makes me crave nicotine.” “It should be accompanied by a trigger warning.”
Future prospects	6	5.0	“An extra day of annual leave could encourage staff members to stop smoking.” “Financial recompensation for diagnosing nicotine dependence is important in healthcare settings.”
Non smokers	6	5.0	“I do not have to complete this because I do not smoke.”
Other	10	8.4	

## Data Availability

The data supporting this research are available from the authors on reasonable request.
